# The Biological Function of the Prion Protein: A Cell Surface Scaffold of Signaling Modules

**DOI:** 10.3389/fnmol.2017.00077

**Published:** 2017-03-20

**Authors:** Rafael Linden

**Affiliations:** Laboratory of Neurogenesis, Institute of Biophysics, Federal University of Rio de JaneiroRio de Janeiro, Brazil

**Keywords:** prion protein, neurodegeneration, prion diseases, Alzheimer disease, signal transduction, cell surface, scaffold proteins, signal corruption

## Abstract

The prion glycoprotein (PrP^C^) is mostly located at the cell surface, tethered to the plasma membrane through a glycosyl-phosphatydil inositol (GPI) anchor. Misfolding of PrP^C^ is associated with the transmissible spongiform encephalopathies (TSEs), whereas its normal conformer serves as a receptor for oligomers of the β-amyloid peptide, which play a major role in the pathogenesis of Alzheimer’s Disease (AD). PrP^C^ is highly expressed in both the nervous and immune systems, as well as in other organs, but its functions are controversial. Extensive experimental work disclosed multiple physiological roles of PrP^C^ at the molecular, cellular and systemic levels, affecting the homeostasis of copper, neuroprotection, stem cell renewal and memory mechanisms, among others. Often each such process has been heralded as the bona fide function of PrP^C^, despite restricted attention paid to a selected phenotypic trait, associated with either modulation of gene expression or to the engagement of PrP^C^ with a single ligand. In contrast, the GPI-anchored prion protein was shown to bind several extracellular and transmembrane ligands, which are required to endow that protein with the ability to play various roles in transmembrane signal transduction. In addition, differing sets of those ligands are available in cell type- and context-dependent scenarios. To account for such properties, we proposed that PrP^C^ serves as a dynamic platform for the assembly of signaling modules at the cell surface, with widespread consequences for both physiology and behavior. The current review advances the hypothesis that the biological function of the prion protein is that of a cell surface scaffold protein, based on the striking similarities of its functional properties with those of scaffold proteins involved in the organization of intracellular signal transduction pathways. Those properties are: the ability to recruit spatially restricted sets of binding molecules involved in specific signaling; mediation of the crosstalk of signaling pathways; reciprocal allosteric regulation with binding partners; compartmentalized responses; dependence of signaling properties upon posttranslational modification; and stoichiometric requirements and/or oligomerization-dependent impact on signaling. The scaffold concept may contribute to novel approaches to the development of effective treatments to hitherto incurable neurodegenerative diseases, through informed modulation of prion protein-ligand interactions.

## Introduction

The prion protein, often referred to as *cellular* prion protein (PrP^C^) was discovered amid studies of transmissible spongiform encephalopathies (TSEs), such as Creutzfeldt-Jakob Disease, a low-prevalence, mostly sporadic, fatal and still incurable neurodegenerative disease (Takada and Geschwind, 2013). Since the 1980s evidence has accumulated that these conditions are associated with the misfolding, aggregation, replication and spread of abnormal conformers of PrP^C^, in line with the concept of a *protein-only, infectious particle* which originated the sobriquet *prion* for the anomalous conformer (Prusiner, 1984).

Whereas misfolding of PrP^C^ is usually considered the major, if not indispensable requirement for neurodegeneration in TSEs, experimental work indicated that the normal PrP^C^ conformer serves as a binding site for diffusible Aß peptide oligomers (AßO) in the course of Alzheimer’s Disease (AD; Um and Strittmatter, 2013; Laurén, 2014; Kostylev et al., 2015). The AßO are deemed the major toxic species associated with AD, and accumulate as a consequence of disregulated proteolytic cleavage of the amyloid precursor protein (APP; Lambert et al., 1998; Walsh and Selkoe, 2007; Ferreira and Klein, 2011).

The involvement of PrP^C^ in both TSEs and AD renewed and amplified the interest in this protein, that holds important clues towards the understanding of the pathogenesis, as well as the discovery of novel therapies for both those neurodegenerative diseases. Progress in this direction, however, suffers from controversies over functional properties of the prion protein, the corruption and/or loss of which are likely relevant to both TSEs and AD. The current review compares functional properties of PrP^C^ with those of scaffold proteins involved in the organization of intracellular signal transduction pathways, in support of the hypothesis that the biological function of the prion protein is that of a cell surface scaffold protein (Linden et al., 2008, 2012, 2017).

## Association of the Prion Protein with Both Transmissible Spongiform Encephalopathies and Alzheimer’s Disease

The course of the various types of TSEs (also known as *Prion Diseases*, henceforth abbreviated PrDis) involves the progressive cooptation and misfolding of PrP^C^ molecules from an initial template of abnormal prions (Colby and Prusiner, 2011). Knowledge is still fragmentary as to the kinetics of aggregation and progressive growth of prion oligomers, the ensuing compaction of protease-resistant, insoluble deposits of abnormal conformers of PrP^C^, as well as the conditions that lead to their occasional organization as amyloid proper (Morris et al., 2009; Eichner and Radford, 2011; Corsaro et al., 2012; Wang et al., 2016). Also the purported toxic species are a matter of debate (Bucciantini et al., 2002; Silveira et al., 2005; Guerrero-Muñoz et al., 2014), as are hypotheses concerning the requirement for ancillary pathogenic factors (Cordeiro and Silva, 2005; Manuelidis, 2013). That the presence of the prion protein is required for the course of PrDis was, however, made clear by early experiments, in which neither the spread of abnormal conformers, nor the pathological hallmarks of PrDis were found in the brains of PrP^C^-null mice infected with extracts of diseased tissue (Büeler et al., 1993).

On the other hand, experimental studies showed that PrP^C^ may bind oligomers of Aß peptide (AßO) and mediate signal transduction induced by the latter (Laurén et al., 2009; Nygaard and Strittmatter, 2009; Chen et al., 2010; Barry et al., 2011; Bate and Williams, 2011a; Larson et al., 2012; Ganzinger et al., 2014; Laurén, 2014). Notably, however, the reputed role of the prion protein as a receptor for AßO is not exclusive (Balducci et al., 2010; Calella et al., 2010; Cissé et al., 2011; Forloni and Balducci, 2011). Several other molecules interact with AßO in both neurons and glial cells (Mucke and Selkoe, 2012; Kam et al., 2014; Ferreira et al., 2015; Yu and Ye, 2015). Importantly, the composition of the preparations of AßO employed in distinct experimental studies is quite variable (Mucke and Selkoe, 2012; Ferreira et al., 2015), and for example, whereas the Frizzled receptor preferentially binds oligomers of low molecular weight and/or monomeric Aß peptide, higher molecular weight oligomers bind the prion protein (Magdesian et al., 2008; Kostylev et al., 2015). This probably explains the multitude of putative neurotoxic AßO receptors, albeit selective oligomer-receptor interactions may legitimately represent the progressive effects of the variegated and evolving Aß peptide aggregates present in the brains of patients along the course of AD (Amieva et al., 2005; Mucke and Selkoe, 2012; Villemagne et al., 2013; Bernard et al., 2014; Alzheimer’s Association, 2015).

## The Quest for the Function of the Prion Protein

The production of the first *Prnp*-null mouse in the 1990s (Büeler et al., 1992) triggered major advances in the field, as it allowed proof that PrP^C^ was required for progression of PrDis in the mouse brain (Büeler et al., 1993). In turn, the report that those mice developed normally and showed no overt behavioral or immunological defects (Büeler et al., 1992), depreciated somewhat the search for functional properties of the normal conformer of PrP^C^. The acme of such dismissal may well be a bold proposal that PrP^C^ has no function, and that its conserved amino acid sequence was naturally selected as a consequence of the deadly effects of mutations (Prcina and Kontsekova, 2011).

Still, the last 15 years witnessed growing interest in the functional properties of PrP^C^, based on analyses of mice devoid of its coding gene* Prnp*, transgenic animals harboring various mutated or partially-deleted forms of PrP^C^, or *Prnp*-overexpressing mice, as well as experimental cross-linking of PrP^C^ with antibodies, engagement with binding peptides or glycosaminoglycans (GAGs), and interference with plasma membrane lipids, eventually accompanied by simultaneous activation of other membrane proteins (reviewed in Martins et al., 2002; Westergard et al., 2007; Linden et al., 2008, 2012; Málaga-Trillo and Sempou, 2009; Martin-Lannerée et al., 2014; Onodera et al., 2014).

It appears to be settled that the prion protein mediates mechanisms of neuroprotection (Martins et al., 2010; Biasini et al., 2012; Béland and Roucou, 2014). However, contributions of PrP^C^ have been reported also in immune responses, energy metabolism, cancer, and stress conditions in general (Linden et al., 2008; Li et al., 2011; Mariante et al., 2012; Martin-Lannerée et al., 2014; Onodera et al., 2014; Bakkebø et al., 2015; Zeng et al., 2015). Often, each such demonstration was heralded as the *bona fide* physiological function of PrP^C^, claims of which range from the systemic level, such as the consolidation of memory, through cellular, such as cytoprotection, down to the subcellular level, such as the homeostasis of copper (Table 1). Nonetheless, the vast majority of the corresponding data, actually disclosed either circumstantial *contributions* to cellular or systemic processes, or *phenotypes*, in some cases specific to certain mouse strains, rather than the unraveling of an unambiguous function at the molecular level (Ashburner et al., 2000; Dessimoz and Škunca, 2017).

**Table 1:** **Keywords to *processes* at the molecular, cellular and system levels, upon which presumptive *functions* have been ascribed to the prion protein**.

Level	Process
Molecular	Homeostasis of copper
	Ion fluxes
	Transport of metabolites
	Redox homeostasis
Cellular	Cell proliferation
	Cell adhesion
	Cell differentiation
	Cell survival
	Cell death
	Neurite outgrowth
	Myelin maintenance
	Synaptic transmission
	Synaptogenesis
	β-amyloid toxicity
	T cell activation
System	Memory
	Sleep
	Embryogenesis
	Inflammation
	Stem cell renewal
	Muscle physiology
	Glucose homeostasis

It is therefore not surprising that current literature pictures the function of PrP^C^ as “unknown”, “unresolved”, “uncertain”, “obscure”, “abstruse”, or “elusive”, among other demeaning terms. Indeed, some of the alleged functions coexist with their opposites. For example, despite substantial agreement that PrP^C^ supports cytoprotection (Liang et al., 2006; Martins et al., 2010; Mehrpour and Codogno, 2010; Santos et al., 2015), proapoptotic effects have also been reported (Paitel et al., 2002; Solforosi et al., 2004; Zhang et al., 2006). Whereas the binding of PrP^C^ to the co-chaperone hop/STI1 triggers neuroprotective signals (Zanata et al., 2002), and the expression of PrP^C^ is associated with enhanced synaptic function (Robinson et al., 2014), binding of PrP^C^ to AßO induces synaptotoxic signals (Nygaard and Strittmatter, 2009). Also, the prion protein reportedly stimulates the proliferation of stem cells (Steele et al., 2006; Santos et al., 2011), but may also shift the phenotype of human embryonic stem cells from self-renewal to differentiation (Lee and Baskakov, 2013).

Granted, methodological differences as well as distinct experimental preparations might explain such contradictory effects. However, the latter are also consistent with a strong cell type- and context-dependency in the behavior of PrP^C^ (Linden et al., 2008; Steele et al., 2009). Such an abundance and variety of functional properties is even more striking considering that the vast majority of mature PrP^C^ molecules are tethered to the outer leaflet of the plasma membrane through a glycosyl-phosphatydil inositol (GPI) anchor (Stahl et al., 1987), and therefore lack an intracellular domain capable of transferring signals from the extracellular environment to the intracellular milieu. Signal transfer involving the prion protein must therefore be conveyed by transmembrane molecules engaged either together with or through PrP^C^. Analysis of such molecular complexes is required to understand the roles of the prion protein in physiological context, as well as its multiple interventions in both health and disease.

Research on PrP^C^-binding partners was originally directed at the identification of a so-called “protein X”, participant in the conversion of PrP^C^ into the scrapie form (Yehiely et al., 1997), or otherwise involved in the formation and propagation of prions (Caughey and Baron, 2006). Over the years a list of putative PrP^C^-binding partners grew out of various approaches (Schmitt-Ulms et al., 2004; Aguzzi et al., 2008; Linden et al., 2008). Several such interactions were validated through compelling biochemical and cell biological procedures, and in each individual case the results were interpreted as evidence for the respective authors’ view of the long sought fundamental function of PrP^C^. In contrast, a number of other putative ligands still lack rigorous confirmation or, often, are unlikely to pair with PrP^C^ in physiological context due to incongruous topologies (Aguzzi et al., 2008; Linden et al., 2008). Nonetheless, even the current consensus around only a handful of strictly validated binding partners allows for the conclusion that PrP^C^ is poised to participate in a variety of combinatorial, multiprotein complexes at the cell surface (Martins et al., 2002, 2010; Linden et al., 2008). The composition of such molecular arrangements is expected to depend on both cell type and context—the former determines the repertoire of binding partners available at the cell surface, whereas the latter modulates their stoichiometry and pattern of activation. The influence of both these factors is further enriched by the rapid and continuous trafficking of PrP^C^ among distinct plasma membrane domains, and the repeated cycles of endocytosis and resurfacing prior to degradation of individual PrP^C^ molecules (Harris, 2003; Prado et al., 2004).

To account for the abundance of cell- and context-dependent, PrP^C^-related roles and phenotypes, as well as the growing list of validated binding partners, we advanced the hypothesis that PrP^C^ functions as a dynamic platform for the assembly of signaling modules at the cell surface, analogous to the scaffold proteins involved in the organization of intracellular signal transduction pathways (Linden et al., 2008, 2012). This theory is further discussed here, in light of three decades of studies that led to the robust characterization of intracellular scaffold proteins (Langeberg and Scott, 2015).

## The Prion Protein as a Cell Surface Scaffold Protein

The current concept of a natural scaffold protein is that of an intracellular, multivalent molecule that binds several members of a signaling pathway leading to a higher order, spatially restricted ensemble, which optimizes downstream signal transfer (Morrison and Davis, 2003; Good et al., 2011; Langeberg and Scott, 2015). Early work suggested that the role of such proteins was limited to the holding of intracellular enzymes in close proximity (Faux and Scott, 1996), but subsequent studies uncovered remarkable structural and functional plasticity (Chen et al., 2005; Brennan et al., 2011; Pan et al., 2012; Smith and Scott, 2013; Barbar and Nyarko, 2014), which helps these molecular platforms regulate spatial, temporal and kinetic properties of signal transduction pathways (Pan et al., 2012). The following sections consider the parallels between the fundamental properties of the prion protein and those of intracellular scaffold proteins.

### Assembly of Multicomponent Signaling Modules

**Figure 1:**
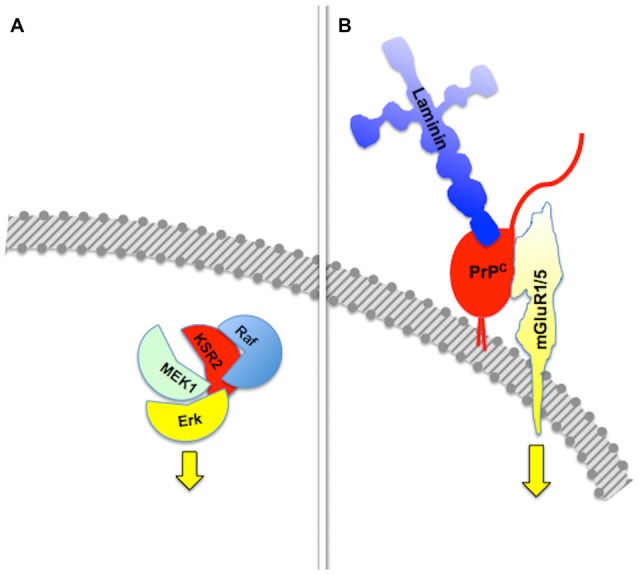
**Assembly of multicomponent signaling modules.** This and the following figures depict multiprotein signaling modules assembled around a scaffold protein (shown in red); the diagram to the left of the vertical bar portrays an intracellular signaling module organized by a consensual scaffold protein, and the scheme to the right represents a cell surface signaling module scaffolded by the prion protein. Yellow arrows indicate output signals from the scaffolded complex. Except where explicitly indicated, the form of each drawing or their juxtaposition indicate binding only, and do not imply either structural or spatial arrangements. **(A)** KSR2-scaffolded MAP kinase cascade based on Kolch (2005). **(B)** PrP^C^-scaffolded, mGluR1/5-mediated, laminin γ1-induced signaling module based on Beraldo et al. (2011).

The basic property of a scaffold protein was originally seen as the offer of a molecular architecture that organizes an intracellular signaling cascade, through the binding of several sequential members of a defined pathway (Pawson and Scott, 1997; Whitmarsh and Davis, 1998). Consistent with this fundamental property, defined sets of molecules among those known to interact with the prion protein compose functional assemblies with specific signaling properties (Figure 1).

Group I metabotropic receptors mGluR1 and mGluR5 belong to a subclass of receptors for the ubiquitous neurotransmitter glutamate (Ferraguti et al., 2008; Ribeiro et al., 2010). These receptors, originally identified as potential ligands of the prion protein in a PrP^C^-baited phage display screen and validated through biochemical experiments (Beraldo et al., 2011), are required to trigger intracellular phospholipase C (PLC)-mediated calcium signals induced in hippocampal neurons by the binding of PrP^C^ to a peptide from the γ1 chain of the extracellular matrix protein Laminin (Ln-γ1; Graner et al., 2000; Beraldo et al., 2011). Signaling through this pathway induces neuritogenesis in both isolated hippocampal neurons and PC12 cells (Beraldo et al., 2011). In turn, the α7 type of nicotinic acetylcholine receptor (α7nAChR) was also shown to bind PrP^C^ (Beraldo et al., 2010), and this interaction was required to trigger calcium influx, the activation of both protein kinase A and Erk, and trophic responses in isolated hippocampal neurons following the binding of the cochaperone hop/STI1 to PrP^C^ (Zanata et al., 2002; Lopes et al., 2005; Beraldo et al., 2010).

Somewhat similar results were reported following experiments done with dorsal root ganglion (DRG) neurons (Santos et al., 2013), where both hop/STI1 and Ln-γ1 induced calcium responses and axon elongation. Here again, the responses triggered by Ln-γ1:PrP^C^ interaction were mediated by mGluR1/5. Distinct from hippocampal neurons, however, signals triggered by hop/STI1:PrP^C^ binding in DRG neurons were traced to the TRPC family of transient calcium receptor channels (Ramsey et al., 2006), rather than to α7nAChR (Santos et al., 2013). It is not known whether the latter result is due to direct PrP^C^:TRPC binding, or to an indirect cell surface interaction, but the differing results reported in neurons either from the central (CNS) or peripheral (PNS) nervous system (Beraldo et al., 2010; Santos et al., 2013) are consistent with the aforementioned cell- and context-dependence of PrP^C^-mediated signal transduction. Importantly, evidence was shown for DRG neurons, but not for hippocampal neurons, of synergism between the hop/STI-1:PrP^C^ and Ln-γ1:PrP^C^ effects, as well as of simultaneous occupation of binding sites in PrP^C^ by both ligands (Santos et al., 2013), supporting the view that PrP^C^ scaffolds multiple molecules at the cell surface, however depending on both cell type and context.

### Crosstalk of Scaffolded Signaling Pathways

In contrast with the early idea of an exclusive intracellular scaffold protein for each defined set of signaling partners, subsequent work disclosed extensive crosstalk among scaffolded signaling networks (Pan et al., 2012). Thus, scaffold proteins such as β-arrestins or axin may each engage multiple signaling cascades (Luo and Lin, 2004; Dard and Peter, 2006). In turn, distinct scaffold proteins may organize the same set of signaling intermediates, such as the Raf-MEK-Erk kinase pathway (Pan et al., 2012; Witzel et al., 2012). In addition, scaffold proteins promote interactions of various signaling modules (Kolch, 2005; Dhanasekaran et al., 2007; Pan et al., 2012), and are subject to regulatory feedback control (Good et al., 2011; Witzel et al., 2012).

**Figure 2:**
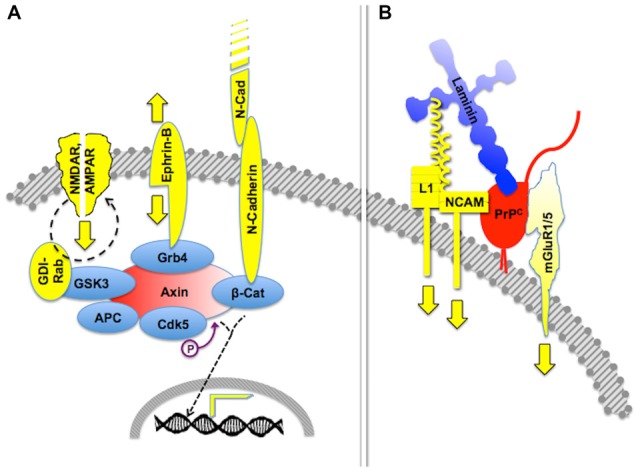
**Crosstalk of multicomponent signaling modules.** The drawings represent multiple signaling modules that may be organized by either an intracellular scaffold protein or the cell surface cellular prion protein PrP^C^. **(A)** Intracellular protein Axin functions as a major scaffold for components of synapses, which include interactive glutamate receptors of the NMDA and AMPA types, bidirectional signaling by ephrin-B, and adhesive interactions through N-cadherin, the latter of which modulates gene transcription through β-catenin. Based on Wei et al. (2010) and Chen et al. (2013). **(B)** Expected crosstalk of Laminin (Ln)-induced signals, transferred to the intracellular milieu through PrP^C^-scaffolded mGluR1/5-, NCAM- and L1-mediated pathways. Based on Nielsen et al. (2010) and Beraldo et al. (2011).

Certain signaling modules based on PrP^C^ display similar features. Thus, PrP^C^ binds several isoforms of the neural cell adhesion molecule (NCAM; Schmitt-Ulms et al., 2001; Slapšak et al., 2016), a cell surface-adhesion molecule of the immunoglobulin superfamily. NCAMs are widely expressed in many tissues and are especially abundant in the nervous system, where they mediate both neural histogenesis and plasticity through homophilic cell-cell interactions (Edelman, 1986; Rutishauser and Landmesser, 1996). Upon binding to PrP^C^, NCAM is recruited to lipid rafts, which facilitates interaction with the soluble Fyn protein kinase, thus leading to intracellular signaling (Santuccione et al., 2005). In addition, the extensive network of NCAM-binding cell adhesion molecules, proteoglycans and extracellular matrix molecules (Nielsen et al., 2010) adds an additional layer of complexity to PrP^C^-mediated signaling components containing NCAM. Interestingly, at least one NCAM binding partner, the cell adhesion molecule L1, also binds laminin (Hall et al., 1997), which forms a loop that may amplify the consequences of the PrP^C^-NCAM interaction. Thus, it is expected that the engagement of PrP^C^ by binding to laminin entails cross-linked activation of multiple signaling pathways, through the concurring transfer of transmembrane signals through mGluR1/5, NCAM and L1 (Figure 2).

Importantly, the 37 kDa Laminin Receptor Precursor/Laminin Receptor (LRP/LR) has been identified as an additional binding partner of PrP^C^ (Rieger et al., 1997). Two sets of cognate binding sites were identified in both partners, one of which required mediation of heparan sulfate proteoglycan, and included a heparin-binding site in PrP^C^ (Hundt et al., 2001; Warner et al., 2002). Interestingly, the binding site in LRP/LR for both Laminin and PrP^C^ is the same (Rieger et al., 1999), and the binding sites in PrP^C^ for both LRP/LR and Laminin partially overlap (Linden et al., 2008), which implies an even more intricate arrangement of PrP^C^-mediated, laminin-induced signaling.

### Allosteric Properties of Multiprotein Signaling Modules

Mechanisms of regulation of scaffold proteins and their clients include reciprocal allosteric changes (Pan et al., 2012; Langeberg and Scott, 2015). For example, the scaffold protein Kinase Suppressor of Ras (KSR), which regulates signal transduction through MAPK pathways (Witzel et al., 2012), not only allosterically modulates the activity of its client protein kinases (Langeberg and Scott, 2015), but its own kinase activity is unlocked upon binding to B-RAF, which facilitates downstream phosphorylation of MEK (Brennan et al., 2011; Hu et al., 2011).

**Figure 3:**
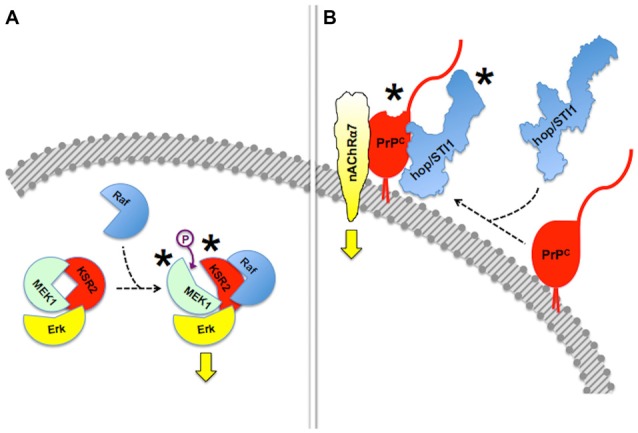
**Allosteric properties of signaling modules.** In this figure, asterisks indicate allosteric events. **(A)** Binding of Raf to scaffold protein KSR2 leads to structural rearrangement in the MEK1 kinase and ensuing activation of Erk. Based on Brennan et al. (2011) and Langeberg and Scott (2015). This simplified diagram does not include dimerization events also indicated by Brennan et al. (2011). **(B)** Reciprocal allosteric changes induced by the binding of hop/STI1 to PrP^C^. Compaction of hop/STI1 and structural remodeling within at least the PrP^C^_143–153_ α-helix may engage the transmembrane signaling proteins LRP/LR and NCAM, both of which bind that domain of PrP^C^, but the binding domains involved in the PrP^C^:nAChRα7 interaction are still unknown. Based on Romano et al. (2009) and Linden et al. (2012).

Reciprocal allosteric effects have also been shown in experiments done with recombinant PrP^C^ and some of its binding partners. Thus, the binding of the co-chaperone hop/STI1 to PrP^C^ induced C-terminal compaction of the former, detected by modeling through small-angle X-ray spectroscopy (SAXS), as well as a slight loss of PrP^C^ α-helical structure, involving at least the PrP^C^_143–153_ (H1) α-helix (Romano et al., 2009; Figure 3). The latter domain of PrP^C^ contains binding sites for both LRP/LR and NCAM (Hundt et al., 2001; Santuccione et al., 2005), which raised the hypothesis that the prion protein may compute signaling triggered by multiple ligands. Interestingly, whereas a PrP^C^-binding hop/STI1 peptide mimicked the full hop/STI1 protein in the induction of several PrP^C^-mediated responses in neurons (Zanata et al., 2002; Lopes et al., 2005), the proliferative effect of hop/STI1 upon glioblastoma cells also depended on the hop/STI1:PrP^C^ interaction, but was not induced by the peptide alone (Erlich et al., 2007; Linden et al., 2012). The latter effect is likely associated with the reciprocal allosteric modulation between hop/STI1 and PrP^C^, and a higher order interaction may involve one or more additional hop/STI1 partners (Linden et al., 2012).

Also consistent with allosteric control of cell surface complexes, is the evidence that a variety of human TSE-related point mutations along the globular domain of PrP^C^ both enhanced the binding of GAGs to the far N-terminal of PrP^C^, as well as unlocked a normally hidden GAG-binding site midway between differing mutations (Yin et al., 2007). These results may signify an impact on signaling properties of LRP/LR, since the interaction of this receptor with one of its target sites in PrP^C^ depends on mediation by a heparan sulfate proteoglycan (Hundt et al., 2001). It should be noted that although PrP^C^ mutations examined in this context were all disease-associated (Yin et al., 2007), several of those correspond to aminoacid residues located either within or close to the binding sites of functionally relevant PrP^C^ ligands such as hop/STI1 (Zanata et al., 2002) or mGluR5 (Haas et al., 2014).

Furthermore, an antibody that targets residues in the α1 and α3 helices of the C-terminal globular domain of PrP^C^ resulted in severe toxicity dependent on the latter’s N-terminal (Sonati et al., 2013). This study pointed to remarkable long-distance interactions along the full extent of PrP^C^. Interestingly, although the set of residues of PrP^C^ that underwent chemical shifts detectable through nuclear magnetic resonance upon antibody binding did not include the N-terminal, they overlapped extensively with the domains involved in the interaction of the prion protein with both Laminin and NCAM (Gauczynski et al., 2001; Santuccione et al., 2005; Sonati et al., 2013). More recent work showed that an engineered GPI-anchored, N-terminal only PrP^C^ molecule (PrP_Δ141–225_, dubbed FTgpi) mimicked the effect of the toxic antibody. Thus, FTgpi bound the endoplasmic reticulum (ER) chaperone Immunoglobulin heavy chain-Binding Protein/Glucose-Regulated Protein 78 (Bip/GRP78), and such binding was followed by sustained ER stress, reduced FTgpi protein/mRNA ratio due to rapid proteolysis, as well as activation of the Protein Kinase R-like ER Kinase (PERK), and cell death (Dametto et al., 2015). Differing, however, from these results, the previous study from the same group did not report any change in the content of full length PrP^C^ upon binding of the toxic antibody (Sonati et al., 2013). Thus, it is not clear whether the toxicity of the latter engages the same mechanisms that link FTgpi with fatal ER stress, or alternatively, depend on interactions of the N-terminal of PrP^C^ at the cell surface. Interestingly, other than its canonical location with the ER, Bip/GRP78 is also found both at the cell surface and in the extracellular medium upon cellular stress (Delpino and Castelli, 2002; Corrigall et al., 2004; Marín-Briggiler et al., 2010; Panayi and Corrigall, 2014; Tsai et al., 2015), therefore potentially subject to scaffolding by an allosterically activated N-terminal domain of PrP^C^.

### Compartmentalization of Scaffolded Signaling Modules

Besides allosteric modulation, the activities of intracellular scaffold proteins are subject to robust regulation by several other mechanisms (Morrison and Davis, 2003; Dard and Peter, 2006). Distribution to selected subcellular domains is required for the spatial and temporal restriction of the activity of signaling modules, as exemplified by the nucleocytoplasmic shuttling of both the yeast Ste5p and mammalian β-arrestin (Mahanty et al., 1999; Wang et al., 2003), or the tethering of KSR to the plasma membrane (Zhou et al., 2002; Ory and Morrison, 2004; Koveal et al., 2012).

**Figure 4:**
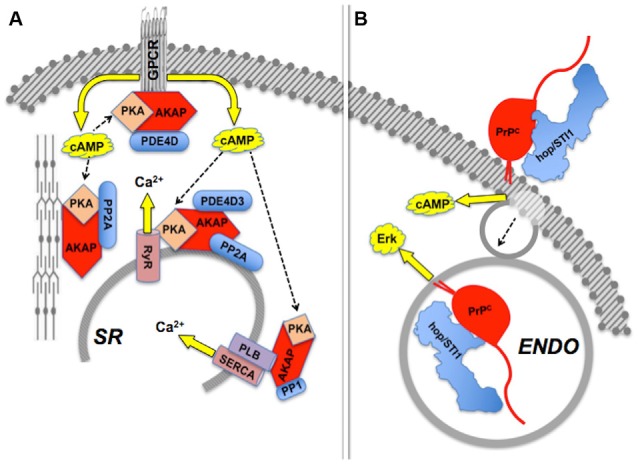
**Compartmentalization of scaffolded signals. (A)** Sets of AKAP-scaffolded client proteins located in distinct compartments lead to distinct intracellular signals mediated by cAMP-PKA activity or calcium fluxes. Modified from Fu et al. (2013). **(B)** Binding of hop/STI1 to PrP^C^ leads to endocytosis-independent signaling through cAMP, and endocytosis-dependent Erk signaling. The PrP^C^-binding clients involved in each case are yet to be determined. Based on Americo et al. (2007) and Caetano et al. (2008).

Effects induced by hop/STI1:PrP^C^ interaction provide an example of compartmentalization of PrP^C^-mediated signaling (Figure 4). The binding of hop/STI1 to PrP^C^ in CNS neurons engages the cAMP-PKA, as well as the Erk signaling pathways (Chiarini et al., 2002; Zanata et al., 2002). Both responses were blocked either by α-bungarotoxin, a specific inhibitor of the α7 nicotinic cholinergic receptor, or by the removal of extracellular calcium, which together with evidence of the binding of PrP^C^ to α7nAChR, implicated this membrane receptor in the PrP^C^-mediated cell responses to hop/STI1 (Beraldo et al., 2010). Nevertheless, the activation of Erk induced by hop/STI1 was abolished by prevention of PrP^C^ endocytosis, while that of the cAMP-PKA pathway persisted (Americo et al., 2007; Caetano et al., 2008). It is likely that the latter results reflect changes in PrP^C^-ligand interactions as the prion protein moves along distinct compartments. One hypothesis to explain these results stems from the changing physicochemical environment along the endocytic pathway. Thus, the concentration of calcium collected via pinocytosis to the lumen of endosomes undergoes an initial rapid decrease compared with the extracellular medium, due to both acidification and the activity of various transporters (Gerasimenko et al., 1998). This strongly affects the binding of several plasma membrane receptors to their cognate ligands (Andersen and Moestrup, 2014). Also, the progressive acidification along the endocytic pathway is, by itself, expected to promote changes in structure and thermodynamic stability of the prion protein (Gu et al., 2003; Zahn, 2003; Chiang et al., 2008; Biljan et al., 2012; Kovač et al., 2016), which in combination with changes in luminal calcium, may modulate the binding of PrP^C^ ligands. Further work directed at the characterization of the dynamic behavior of PrP^C^ ligands, in particular in response to Ca^2+^ levels, is therefore warranted to clarify mechanisms that regulate the compartmentalization of PrP^C^-mediated signal transduction.

In addition to the PrP^C^-ligand interactions *in cis* described above, other studies of both soluble PrP^C^ and its fragments also typify compartmentalized signaling. Thus, various soluble recombinant forms of PrP^C^ bound to and underwent partial LRP/LR-dependent internalization (Gauczynski et al., 2001), protected human neurons from Bax-mediated apoptosis (Bounhar et al., 2001), induced neurite outgrowth and/or synaptogenesis in cultured cerebellar and hippocampal neurons (Chen et al., 2003; Kanaani et al., 2005), and activated monocytes (Krebs et al., 2006; Jeon et al., 2013) and natural killer cells (Seong et al., 2015). Such effects are contingent upon the activation of a variety of intracellular signaling molecules, including PI3-kinase, Erk, cAMP/PKA or PKC (for review see Linden et al., 2008). Although those results were obtained with recombinant PrP^C^, they are consistent with physiological effects of PrP^C^ *in trans*, either through the release of PrP-containing microvesicles (Porto-Carreiro et al., 2005; Robertson et al., 2006; Vella et al., 2008; Wang et al., 2011; Hajj et al., 2013; Ritchie et al., 2013; Berrone et al., 2015; Guo et al., 2015), or as soluble fragments derived from endoproteolysis of PrP^C^ (Béland et al., 2012; Roucou, 2014).

An extended view of PrP^C^-based interactions *in trans* includes the recently disclosed role of the prion protein upon myelin homeostasis, through the specific interaction of its N-terminal flexible tail with the Adhesion G protein-coupled receptor Gpr126 (Adgrg6; Küffer et al., 2016 and see below). Also recently, evidence was shown that a recombinant, soluble PrP^C^ promoted growth cone (GC) motility and extension of neurites, through *in trans* interactions that depend on cell surface PrP^C^ as well as NCAM, both of which are recruited to common sites at the GC plasma membrane, and involve the activation of several downstream signaling pathways (Amin et al., 2016). The latter are analogous to effects triggered by other extracellular ligands of the prion protein, and suggest a physiological role of either soluble or microvesicle-associated PrP^C^ upon neurite outgrowth. A notable requirement for the reported effect *in trans* was the integrity of the soluble PrP^C^ molecule (Amin et al., 2016), which is consistent with long-range allosteric interactions throughout the full extent of the prion protein (Yao et al., 2003; Yin et al., 2007; Christen et al., 2009; Thakur et al., 2011; Sonati et al., 2013; Spevacek et al., 2013). It is, however, not yet known whether the effects of the recombinant PrP^C^ in physiological context may require its location at the surface of extracellular microvesicles.

### Posttranslational Regulation of Scaffolding Properties

Functional regulation of intracellular scaffold proteins also relies upon phosphorylation (Ory and Morrison, 2004; Good et al., 2011; Tacchelly-Benites et al., 2013; Langeberg and Scott, 2015) or ubiquitination (Shenoy et al., 2001). Neither has been so far described for the prion protein, but other posttranslational modifications of native PrP^C^ molecules affect signaling properties.

**Figure 5:**
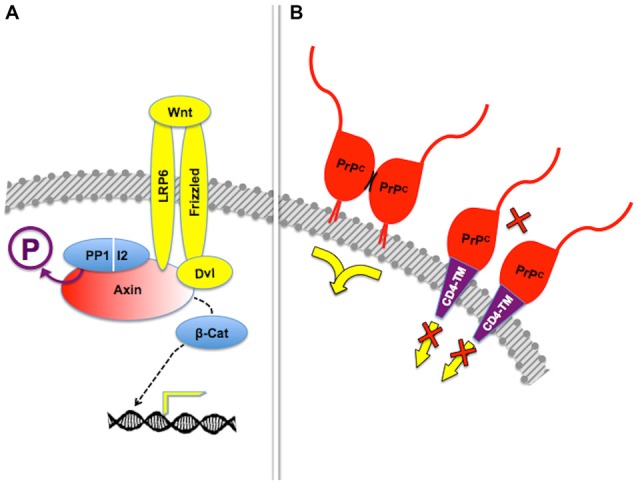
**Posttranslational regulation of scaffolding properties. (A)** Dephosphorylation of the scaffold protein Axin by the PP1-I2 phosphatase complex is required for β-catenin-induced gene expression in response to Frizzled-mediated Wnt signaling. Adapted from Tacchelly-Benites et al. (2013). **(B)** Tetethering of PrP^C^ through the glycosyl-phosphatydil inositol (GPI) anchor, but not through an heterologous transmembrane domain, was needed for protection of neuroblastoma cell lines from cell death induced by the excitotoxin kainate. In this context, it was shown that stress protection depended on dimerization of PrP^C^ (short, curved black lines), but additional molecules required for the transfer of cytoprotective signals have not been identified. Based on Rambold et al. (2008).

The GPI anchor was reported as required for the formation of cell-surface PrP^C^ dimers, which in turn were needed for PrP^C^-mediated protection from cellular stress (Rambold et al., 2008). Consistent with a previous theoretical model (Warwicker, 2000), the short internal hydrophobic domain PrP^C^_113–133_ was identified as the dimerization domain (Rambold et al., 2008; Figure 5). This finding strengthens the notion that posttranslational modifications impart PrP^C^ properties relevant for signal transduction. The GPI anchor is also critical for the trafficking of PrP^C^ along distinct plasma membrane domains (Harris, 2003; Prado et al., 2004), which underlies the above-mentioned dependence of downstream signals on endocytosis of PrP^C^, and in particular for the targeting of the prion protein to lipid rafts (Morris et al., 2006; Taylor and Hooper, 2006; Puig et al., 2014). The latter explains, for example, the recruitment of NCAM towards the preferential location of its intracellular signaling partner, the soluble Fyn kinase (Santuccione et al., 2005), as well as the association of PrP^C^ with reggie/flotillins (Stuermer and Plattner, 2005), which drives both downstream MAP kinase and calcium signals (Stuermer et al., 2004). Recent work also attributes to the GPI anchor an important role in PrP^C^ processing and the shedding of bioactive fragments (Puig et al., 2014).

Furthermore, the composition of the GPI anchor was shown to regulate both the lipid content of membrane microdomains and the localization of PrP^C^ therein (Bate and Williams, 2011b; Bate et al., 2016), with concurring changes in synaptotoxic signaling triggered by cross-linking of PrP^C^ molecules with either AßO or antibodies, and mediated by phospholipase A2 (PLA2; West et al., 2015; Bate et al., 2016). Interestingly, PLA2 has also been identified as a mediator of the release of the APP ectodomain induced by activation of the PrP^C^ ligand mGluR1 (Nitsch et al., 1997). These data are consistent with an important, selective role of the GPI anchor upon the interaction of PrP^C^ with its client proteins.

Another critical postranslational modification of PrP^C^ is the N-linked glycosylation of either one or both target asparagine residues (Turk et al., 1988; Rudd et al., 2002; Lawson et al., 2005). Comparative analysis of PrP^C^ either from the brain or from peripheral blood mononuclear cells (PBMC), showed that the relative content of the unglycosylated and glycosylated forms differed between the two samples (Li et al., 2001). Distinct patterns of sialylation have also been described for PrP^C^ from either brain or spleen (Baskakov and Katorcha, 2016), and possible roles of sialylation upon functional properties of PrP^C^ were discussed (Baskakov and Katorcha, 2016). Other studies indicated that posttranslational modifications produce a collection of differing glycosylated forms of PrP^C^ (Pan et al., 2002), which vary across distinct brain regions (Kuczius et al., 2007b) and change with aging (Goh et al., 2007). Heterogeneous glycosylation is likely to impart selectivity of ligand binding, as suggested both by differential binding to antibodies (Li et al., 2001; Kuczius et al., 2007a) and metal ions (Moudjou et al., 2007), as well as by the differing outcomes of peripheral inoculation of TSE upon experimental prion disease in mice expressing distinct glycosylated forms of PrP^C^ (Cancellotti et al., 2010). Indeed, a coimmunoprecipitation experiment in our lab suggested that interaction of PrP^C^ with the purinergic receptor P2X4R depends on the pattern of glycosylation of PrP^C^ (Carneiro et al., 2016).

### Stoichiometry and Oligomerization in Scaffold-Client Signaling Modules

In early attempts to model the behavior of scaffold proteins, attention was focused on their binding selectivity and their ensuing ability to spatially concentrate sequential components of intracellular signaling modules (Eungdamrong and Iyengar, 2004). Such studies gradually evolved to the matter of stoichiometry of multiprotein assemblies, and the effects of varying concentrations of either the scaffold or their client proteins (Bray and Lay, 1997; Bray, 1998; Levchenko et al., 2000; Heinrich et al., 2002; Locasale et al., 2007; Kholodenko et al., 2010). Pertinent to the matter of stoichiometry, the effects of certain scaffold proteins upon signaling efficacy were traced to their oligomerization (Yablonski et al., 1996; Elion, 2001; Ren et al., 2005; Chen et al., 2008; Gold et al., 2011; Abel et al., 2015; Liu et al., 2016).

A major challenge to a deeper understanding of the scaffolding function is, however, the scarcity of data regarding both kinetic parameters and relative concentrations of signal transducers in confined intracellular domains, which are required for the full understanding of signaling dynamics (Langeberg and Scott, 2015). Still, the stoichiometries of certain scaffold-client complexes have been unraveled. For example, the scaffold protein AKAP79 forms a 2:2:2:2 complex with its client proteins calmodulin, calcineurin and a PKA regulatory subunit (Gold et al., 2011), whereas AKAP18γ forms a 1:2 complex with a PKA regulatory subunit (Smith et al., 2013), and the NOD-like receptor NLRP3 was proposed to form a multimeric, equimolar inflammasome with Caspase-1 through the adaptor protein Apoptosis-associated Speck-like protein containing a CARD (ASC; Lechtenberg et al., 2014). An especially complex case is the postsynaptic density (PSD), which contains large numbers of proteins, including neurotransmitter receptors, adaptor and effector proteins organized in aggregates visible through conventional transmission electron microscopy (Harris and Weinberg, 2012). Importantly, certain differences were reported among the relative concentrations of PSD components in differing areas of the CNS (Cheng et al., 2006; Sheng and Hoogenraad, 2007; Lowenthal et al., 2015; Patrizio and Specht, 2016).

So far, growing interest in ligands of the prion protein has yet to lead to direct analysis of the stoichiometry of the PrP^C^-based signaling modules, and this is, at this time, the least understood among the features discussed herein with respect to intracellular scaffold proteins. Nevertheless, many studies have compared either the phenotypes of mice, or the properties of cells harboring differing contents of PrP^C^. For example, by comparing *Prnp*-KO, WT and *Prnp*-overexpressing mice subject to ischemic injury to the brain, it was reported both that PrP^C^ accumulates at the penumbra of hypoxic damage, and that lack of PrP^C^ is associated with aggravated ischemic injury (McLennan et al., 2004; Weise et al., 2004, 2006; Spudich et al., 2005; Mitsios et al., 2007). Transduction of the *Prnp* gene carried by a recombinant viral vector improved neurological behavior and reduced the volume of cerebral infarction in a rat model of cerebral ischemia (Shyu et al., 2005).

The results above suggest a dose-dependent neuroprotective effect of PrP^C^ against hypoxic-ischemic insults, but its mechanisms are unclear. Enhanced sensitivity to ischemic damage in the absence of PrP^C^ was originally attributed to an impairment of the antiapoptotic phosphatidylinositol 3-kinase/Akt pathway, resulting in enhanced postischemic activation of caspase-3 (Weise et al., 2006). However, mice harboring an increased content of PrP^C^ displayed significantly smaller infarct volumes than wild type, accompanied by a reduction in early postischemic Erk1/2 phosphorylation, whereas no difference was detected in postischemic phosphorylation of Akt (Weise et al., 2008). Recently, the same group reported an increased content of lactate dehydrogenase (LDH), as well as evidence of physical interaction of LDH with PrP^C^, and suggested that LDH may mediate PrP^C^-dependent neuroprotection under low oxygen conditions, although the apparent physical LDH:PrP^C^ interaction was localized to the cytoplasm (Ramljak et al., 2015). Still, in those reports no cell surface partners of PrP^C^ have been associated with the altered intracellular signals, which preclude further consideration of stoichiometry.

Other studies showed that hop/STI1 haploinsufficient mice were more vulnerable to ischemic insult and their astrocytes secreted lower amounts of the cochaperone than wildtype. Significantly, PrP^C^ mediated prevention of ischemic insult by extracellular hop/STI1 (Beraldo et al., 2013). Since neurotrophic signals induced by hop/STI1:PrP^C^ interaction in central neurons depend on α7nAChR (Beraldo et al., 2010), and the latter has been implicated in neuronal resistance induced by either nicotine or melatonin against hypoxia (Hejmadi et al., 2003; Parada et al., 2014), the hop/STI1:PrP^C^:α7nAChR signaling complex may be a major player in neuroprotection against ischemic insults. Interestingly, examples of sexually dimorphic, ischemic brain injury mediated by both hormonal and non hormonal mechanisms (Liu et al., 2009; Herson and Hurn, 2010; Manwani and McCullough, 2011; Fairbanks et al., 2012; Herson et al., 2013; Zuo et al., 2013; Sanches et al., 2015) include the sensitivity of hippocampal neurons to ischemia in PrP^C^-null mice (Sakurai-Yamashita et al., 2005), and evidence has been reported of both sexually-dimorphic α-bungarotoxin binding (Arimatsu et al., 1981; Arimatsu and Seto, 1982) as well as changed content of α7nAChR following prenatal stress (Schulz et al., 2013). These data warrant a critical examination of the stoichiometry of hop/STI1:PrPC:α7nAChR complexes in the context of sensitivity to ischemic insults, especially in view of the variegated homo- and/or hetero-multimeric, cholinergic receptors that may assembled around α7nAChR subunits, as indicated by experimental work with various cell types (Bertrand et al., 2015; Wu et al., 2016).

In contrast with the reports above of an unimodal dose-response relationship between cell responses and the content of PrP^C^, differing results were reported as to the sensitivity to ischemic damage of transgenic *Prnp*-overexpressing mice (Spudich et al., 2005; Weise et al., 2006), and several experimental models failed to conform to a regular dose-dependent effect among mice harboring variable amounts of PrP^C^ (Coulpier et al., 2006; Jouvin-Marche et al., 2006; Terra-Granado et al., 2007; Lobão-Soares et al., 2008; Steele et al., 2009; Rial et al., 2012; Alfaidy et al., 2013). In several such cases, it appears that either up- or downregulation of PrP^C^ may induce cellular dysfunction, and again the effect depends on both cell type and context. For example, the recently described dose-response curve of the neuritogenic effect of a soluble recombinant PrP^C^ upon GCs of hippocampal neurons was clearly biphasic (Amin et al., 2016). Results as such strengthen the need for studies of the stoichiometry of PrP^C^-ligand complexes.

**Figure 6:**
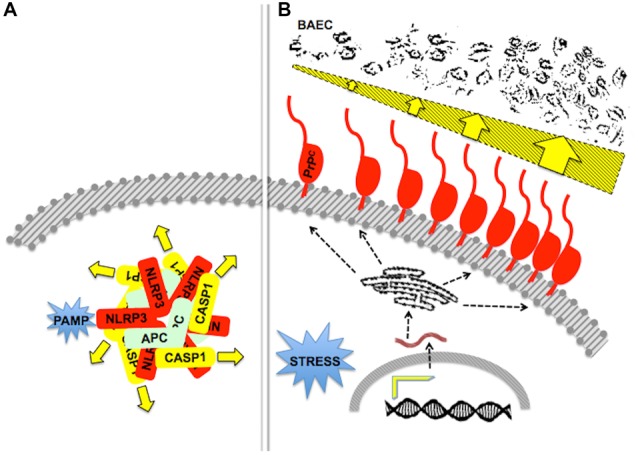
**Stoichiometry and/or oligomerization in scaffold-client signaling modules. (A)** A diagram of the pathogen-associated molecular pattern (PAMP)-activated, NLRP3-scaffolded inflammasome, formed by an equimolar associationof the latter with ASC and caspase-1. The drawing depicts a pentameric arrangement, but the actual stoichiometry is still unknown. Adapted from Lechtenberg et al. (2014). **(B)** The drawing represents the effect of an increased content of PrP^C^ upon the killing of primary bovine aortic endothelial cells (BAEC) by neutrophils. Either inflammatory of behavioral stress induced increased gene expression and a higher content of PrP^C^ at the surface of mouse neutrophils, which was associated with increased neutrophil cytotoxicity towards BAEC (dark profiles at the top). Adapted from Mariante et al. (2012). Effectors of cytotoxicity are still unknown.

In a distinct experimental setting, we showed that the expression of the the *Prnp* gene, as well as the content of PrP^C^ at the cell surface of mouse neutrophils, are selectively augmented by both inflammatory and behavioral stress, as a response mediated by a combination of serum TGFβ and glucocorticoid (Mariante et al., 2012). The increased content of PrP^C^ endowed neutrophils with enhanced peroxide-dependent cytotoxicity toward endothelial cells (Figure 6), the mechanism of which is currently unknown. Studies of the stoichiometry of PrP^C^-dependent signaling complexes in immune cells may thus contribute to the understanding of neurodegenerative events (Beckman and Linden, 2016), in particular those mediated by neutrophils which have recently been implicated in the pathogenesis of AD (Zenaro et al., 2015).

In parallel, several lines of evidence indicate that clustering or oligomerization of native PrP^C^ affect normal signal transduction. Various cellular responses were induced by cross-linking of PrP^C^ with antibodies (Mouillet-Richard et al., 2000; Hugel et al., 2004; Solforosi et al., 2004; Pantera et al., 2009; Tomasi, 2010; Shi et al., 2013), as for example, the association of PrP^C^ with microdomain-forming reggie/flotillin proteins, followed by recruitment of other transmembrane proteins and soluble intracellular protein kinases, leading to downstream signaling (Stuermer et al., 2004).

Native prion protein may likewise form dimers (Priola et al., 1995; Meyer et al., 2000), and in contrast with an earlier report that a recombinant protein failed to exhibit *in vitro* monomer-dimer equilibrium (Meyer et al., 2000), recombinant PrP^C^ was shown to dimerize in solution at room temperature and upon crystalization, through domain swapping and rearrangement of disulfide bonds (Knaus et al., 2001). Copper ions at substoichiometric concentrations also induced self-association of PrP^C^ molecules* in vitro*, without detectable conformational changes in the globular domain (Wells et al., 2006). Furthermore, adding to the above mentioned stress-protection effect of dimeric PrP^C^ *in cis* (Rambold et al., 2008), homophilic interaction of PrP^C^ molecules *in trans* were shown to impart cell adhesion properties particularly important for embryonic development in zebrafish (Málaga-Trillo et al., 2009).

Due to the evidence that the infectivity of abnormal conformers of the prion protein is associated with aggregation (Silveira et al., 2005), functional characterization of PrP^C^ oligomers have usually been limited to their putative role as the basic components of pathogenic prions (Masel et al., 2005; Pan et al., 2005; Zhang et al., 2007; Gerber et al., 2008; Kaimann et al., 2008; Lee et al., 2010; Hafner-Bratkovič and Jerala, 2011; Hafner-Bratkovič et al., 2011; Apostol et al., 2013; Huang et al., 2013; Yu et al., 2016). Nonetheless, there is growing interest in physiological consequences of PrP^C^ dimerization, such as their trafficking to the cell surface, endoproteolysis and shedding of soluble fragments with cytoprotective activity (Yusa et al., 2012; Roucou, 2014), all of which may be subject to stoichiometry-dependent multicomponent assemblies of PrP^C^ and its ligands. This subject clearly needs further attention to allow better understanding of PrP^C^-dependent cell signaling and its consequences upon physiology and behavior.

## Corruption of Prion Protein-Mediated Signaling and the Scaffold Hypothesis in Neuropathology

Mutations and polymorphisms in several members of the AKAP family of intracellular scaffold proteins, such as AKAP12, Ezrin and Merlin have been linked to hyperplastic syndromes and cancer (Poppinga et al., 2014; Han et al., 2015; Petrilli and Fernández-Valle, 2016), while other family members, such as Myospryn and AKAP9, have been associated with skeletal muscle and cardiovascular diseases (Tsoupri and Capetanaki, 2013; Diviani et al., 2016). In particular, a targeted mutation analysis has linked the Long-QT Syndrome (LQTS) to a single missense mutation in AKAP9, which disrupts its binding to a slowly activating cardiac potassium channel (I_Ks_), thus preventing proper cAMP-dependent regulation of the latter, and leading to delayed repolarization of the ventricular action potential (Chen et al., 2007). The latter is a compelling example of the specific requirement of the scaffold-client interaction for maintaining a defined physiological condition. Robust, albeit less precise, genotype-phenotype correlations were inferred for other intracellular scaffold proteins and provisionally traced to scaffold-client interactions, such as the association of severe obesity with rare variants of KSR2, a member of the KSR family (Pearce et al., 2013), and that of certain transcripts of the dystrophin gene with cognitive impairment in a subset of muscular distrophy patients (Daoud et al., 2009; Desguerre et al., 2009; Taylor et al., 2010; Constantin, 2014; Molza et al., 2015). Further work is, however, warranted to reach a similar mechanistic understanding of scaffold corruption associated with mutations in either KSR or Dystrophin, as is the case of the AKAP9:I_Ks_ interaction associated with LQTS.

An analogous hypothesis of scaffold corruption applies to PrP^C^. Thus, the group I metabotropic glutamate receptor mGluR5 reportedly cooperates with PrP^C^ for both AßO binding and toxicity Um and Strittmatter, 2013; Hu et al., 2014). AßO induced cell-surface clustering of PrP^C^ (Caetano et al., 2011), while an mGluR5-selective negative allosteric modulator had a protective effect against both cognitive loss and the accumulation of neuropathological Aß oligomers and plaques in a transgenic AD mouse model (Hamilton et al., 2016). These results are consistent with the evidence for a pathogenic role of the PrP^C^:mGluR5 interaction, which may be linked to disruption of PrP^C^:mGluR5 stoichiometry. In addition, recent studies showed that the co-chaperone hop/STI1 has protective effects upon AßO toxicity, through direct interaction with the PrP^C^-α7nAChR complex (Ostapchenko et al., 2013), and evidence has been reported of a crosstalk between intracellular signaling induced by either AβO or the Ln-γ1 peptide through the PrP^C^-mGluR5 complex in both primary neuron cultures and cell lines (Beraldo et al., 2016). These data implicate at least two extracellular and two transmembrane ligands of PrP^C^ in a cell-surface complex involved in both neurodegenerative and neuroprotective signaling associated with AD.

On the other hand, the recently disclosed interaction *in trans* of the N-terminal flexible tail of PrP^C^ with the Adhesion G protein-coupled receptor Gpr126 was shown to favor myelination of peripheral axons through an increase in the levels of cAMP in Schwann cells, which likely explains the demyelinating polyneuropathy that affects aging PrP^C^-null mice (Küffer et al., 2016). Whereas possible roles of other PrP^C^-interacting molecules have not been examined, it is noteworthy that another known ligand of Gpr126 likewise involved in myelin homeostasis is Laminin-211 (Petersen et al., 2015), which bears the PrP^C^-interacting Laminin γ1 chain (Graner et al., 2000; Beraldo et al., 2011). These data raise the hypothesis of the operation of a signaling complex involving PrP^C^-laminin 211 binding *in cis*, and both PrP^C^- and Laminin 211-Gpr126 *in trans*, in both the physiological control of peripheral nerve myelination and in demyelination conditions.

The prevailing view that TSEs are caused by an exclusive gain-of-toxic function of the scrapie form of the prion protein, has often been challenged by an alternative view that loss-of-function of PrP^C^ is likely to play a role in such diseases (for review see Leighton and Allison, 2016). The latter has historically been dismissed due to the lack of major neurological signs in PrP^C^-null mice (Büeler et al., 1992). Even the evidence of preclinical downregulation of PrP^C^ in several disease models was discussed basically as a possible neuroprotective event, on the grounds that it would provide less substrate for conformational conversion and thus for disease progress (Mays et al., 2014). In fact, gain- and loss-of-function components are not mutually exclusive, and the abundant evidence for neuroprotective effects of PrP^C^ (Zamponi and Stys, 2009; Martins et al., 2010; Biasini et al., 2012; Onodera et al., 2014; Zeng et al., 2015) concurs with the hypothesis that the early and robust loss of PrP^C^ may be involved in the pathogenesis of TSEs. In turn, whereas PrP^C^ has been identified as a pathogenic receptor for AβO in models of AD, binding of the prion protein to hop/STI1 also mediates neuroprotection against AβO neurotoxicity (Ostapchenko et al., 2013), which reinforce the interest in physiological properties of PrP^C^.

## Conclusion and Further Directions

So far, the reported physiological roles of PrP^C^ cannot be reduced to any intrinsic function beyond its ability to bind other molecules required to either overcome the lack of a transmembrane domain in the dominant form of PrP^C^, or to bridge *in trans* cell-cell interaction. Still, most attempts at understanding the Janus-faced behavior of the prion protein in various circumstances have led investigators to concentrate on effects of either the engagement or ablation of PrP^C^, or else to address single PrP^C^ partners. This has usually led to equating the elusive physiological function of PrP^C^ to its role in a particular process or phenotype. Contrary to such a restricted approach, current evidence supports the hypothesis that the functional properties of PrP^C^ are based on its ability to serve as a hub for a large variety of multicomponent signaling modules, with widespread consequences for both physiology and pathology.

The data reviewed above highlight a striking resemblance of both the behavior of PrP^C^ and that of intracellular signaling scaffold proteins. Similar to the latter, the prion protein displays the following properties (Figures 1–6): (a) ability to recruit spatially restricted sets of binding molecules involved in specific signaling; (b) mediation of the crosstalk of signaling pathways; (c) reciprocal allosteric regulation with its partners; (d) compartmentalized responses; (e) dependence of signaling properties upon posttranslational modification; and (f) stoichiometric requirements and/or oligomerization-dependent impact on PrP^C^-dependent effects. These features, added to the widely recognized pleiotropism of PrP^C^, are consistent with our view that the prion protein functions as a scaffold protein, which helps the assembly of various cell type- and context-specific, multicomponent signaling modules at the cell surface (Linden et al., 2008, 2012, 2017).

The recognition of PrP^C^ as a scaffold protein appears to be the closest to philosophical concepts of *biological function*, which imply an unambiguous, unconditional, generalized property of a biological unit (Cummins, 1975; Griffiths, 1993; Diaz-Herrera, 2006; Seringhaus and Gerstein, 2008). Rather than concentrating on any selected, individual binding partner of PrP^C^, such a concept recommends a wider, systemic approach to the variety of signaling modules scaffolded by the prion protein in either physiological or pathophysiological contexts. In view of the failure of several clinical trials directed at either the TSEs or AD (Stewart et al., 2008; Gauthier et al., 2016), this approach may help devise a novel rationale to the development of effective therapeutic options for such refractory neurodegenerative conditions.

## Author Contributions

RL is the sole author and fully responsible for this article.

## Conflict of Interest Statement

The author declares that the research was conducted in the absence of any commercial or financial relationships that could be construed as a potential conflict of interest.
